# Atrial Fibrillation and Cryptogenic Thromboembolic Events

**DOI:** 10.5935/abc.20180141

**Published:** 2018-08

**Authors:** Benhur Davi Henz, Luiz Roberto Leite

**Affiliations:** Instituto Brasília de Arritmias - Hospital do Coração do Brasil (Rede Dor / São Luiz), Brasília, DF – Brazil

**Keywords:** Atrial, Fibrillation, Stroke, Thromboembolism, Foramen Ovale,Patent

Annual stroke rates are extremely high, affecting around 15 million individuals worldwide, generating major public health and economic impact. Approximately 25% of stroke cases do not have a determined etiology, thus being denominated cryptogenic stroke (CS).^1^ Cryptogenic strokes do not have a definite cause; their identification occurs by exclusion, when they are not attributable to definite cardioembolism, large-vessel atherosclerosis of and small-vessel disease, despite extensive vascular, cardiac or serological investigation.^2^

CS rates vary significantly, depending on the degree of diagnostic investigation. Considering that most CS cases have an embolic origin, a new terminology has been recently created for non-lacunar cryptogenic ischemic strokes: “Embolic Stroke of Undetermined Source”.^3^

Approximately one-third of patients with CS have a new ischemic episode in 10 years,^4^ of which 63% are once again classified as cryptogenic.^5^ Possible causes for this recurrence, despite the primary event, are paroxysmal atrial fibrillation (AF), arterial thromboembolism, patent foramen ovale, structural heart disease or less common etiologies, such as thrombophilias. AF detection after a CS or ESUS offers the opportunity to reduce the risk of stroke recurrence by prescribing an oral anticoagulant.^6^ Without this diagnosis, the treatment for CS and ESUS consists only of platelet antiaggregation.^7^

## Detection of subclinical atrial fibrillation in cryptogenic and embolic stroke of undetermined source

The use of long-term monitoring dramatically improved the ability to detect short, rare, and asymptomatic AF periods in stroke patients. The EMBRACE study evaluated 572 patients with ischemic stroke in the last 6 months, with no AF diagnosis, with randomization for 30-day continuous monitoring (287 patients) vs. 24-hour Holter (285 patients).

The AF detection rates (> 30 seconds) were 16.1% in the long-term monitoring group vs. 3.2% in the Holter group.^8^ Similarly, when Implantable Monitors (IM) were used, as in the CRYSTAL-AF (Cryptogenic Stroke and underlying Atrial Fibrillation) study, AF detection rates using IM were higher than the standard detection rates during a long-term follow-up: 8.9%, 12.4% and 30% vs. 1.4%, 2.0% and 3% in the period of 6, 12 and 36 months.^9^

In this issue, Sampaio et al.^10^ published an article on the evaluation of a continuous monitoring device (PoIP) when compared to 24-hour Holter in the diagnosis of atrial arrhythmias in patients with and without stroke, or transient ischemic attack (TIA), and without AF. Episodes of AF were detected in the group of patients with a history of stroke / TIA in 23.1% of patients in the PoIP group and in 3.8% of patients in the Holter group. Lower recording times were also observed in the first 24 hours in the PoIP group vs. Holter group. Atrial tachycardia rates were higher in patients in the stroke group when compared to controls. Significant loss of signal was observed in the PoIP group, of 11.4% due to network instability and different types of signal-sending technology, GPRS vs. 3-4G.

Even with a limited number of patients, the incidence of AF was higher in the long-term monitoring group, although it did not reach statistical significance. However, for this type of monitoring, we need to improve the quality of data transmission, the stability of networks and the technologies used for sending and receiving signals, aiming at lower losses and better quality of the received data.

## Association between atrial fibrillation, cryptogenic and embolic stroke of undetermined source

Recently, several studies have evaluated the association of atrial tachyarrhythmias diagnosed in implantable devices with the risk of thromboembolic events. The MOST study^11^ showed that the detection of periods > 5 minutes of atrial heart rate > 220 bpm was associated with a six-fold increase in the risk of AF and a 2.8-fold increase in the risk of death or stroke in these patients with AF. The TRENDS study^12^ showed that patients with episodes of AF / AT > 5.5 hours / day had an increased risk of thromboembolism (hazard ratio = 2.2), when compared to those with AF / AT burden of zero. Similarly, the ASSERT study demonstrated that the presence of atrial heart rate > 190 bpm for a period of time > 6 minutes was associated with a 5.6-fold increase in the development of AF and 2.5-fold increase in new episodes of stroke or systemic thromboembolism.^13^ A more recent analysis of this study showed that high-frequency atrial episodes lasting > 24 hours increased the risk of ischemic stroke and systemic embolism to 3.1%/year – a risk comparable to that of clinical AF.^14^

Although the high atrial rate with an increased number of embolic episodes is well documented, the temporal and causal association require further elucidation. A sub-analysis of the TRENDS study demonstrated the presence of tachyarrhythmias prior to the embolic event in only 50% of the patients; 73% of them did not have tachyarrhythmias in the 30-day period before the embolic event. Also, the ASSERT study corroborated the results by showing AF rates in 51% of patients with thromboembolism, but only 8% of them had AF in the 30-day pre-stroke period.^15^ The evaluation of these studies suggest that the presence of AF could be simply a marker of thromboembolic risk and be indirectly associated with the occurrence of thromboembolism through a more complex mechanism than the previously expected one.^16^
